# Newborn Genetic Screening—Still a Role for Sanger Sequencing in the Era of NGS

**DOI:** 10.3390/ijns9040067

**Published:** 2023-12-07

**Authors:** Silje Hogner, Emma Lundman, Janne Strand, Mari Eknes Ytre-Arne, Trine Tangeraas, Asbjørg Stray-Pedersen

**Affiliations:** Norwegian National Unit for Newborn Screening, Division of Paediatric and Adolescent Medicine, Oslo University Hospital, 0424 Oslo, Norway; emlund@ous-hf.no (E.L.); jstran@ous-hf.no (J.S.); maytre@ous-hf.no (M.E.Y.-A.); ttangera@ous-hf.no (T.T.); astraype@ous-hf.no (A.S.-P.)

**Keywords:** newborn screening, dried bloodspots, extraction, Sanger sequencing

## Abstract

In the Norwegian newborn screening (NBS) program, genetic testing has been implemented as a second or third tier method for the majority of NBS disorders, significantly increasing positive predictive value (PPV). DNA is extracted from dried blood spot (DBS) filter cards. For monogenic disorders caused by variants in one single gene or a few genes only, Sanger sequencing has been shown to be the most time- and cost-efficient method to use. Here, we present the Sanger sequencing method, including primer sequences and the genetic test algorithms, currently used in the Norwegian newborn screening program.

## 1. Introduction

In Norway, all newborns are offered a screening test through the national newborn screening (NBS) program, and testing is centralized to one single laboratory. Currently, the screening program includes 26 rare, treatable disorders. These include 21 inborn errors of metabolism (IEMs) [1], congenital hypothyroidism (CH), congenital adrenal hypoplasia (CAH), cystic fibrosis (CF) [2], T cell deficiency/severe combined immunodeficiency (SCID) [3], and spinal muscular atrophy (SMA). Among the ca. 55,000 children born each year, 50–60 cases (approximately 1:1000) are identified with one of these 26 disorders. The majority of infants are pre-symptomatic at referral, and early therapeutic intervention is critical to prevent organ damage and/or death. Early detection and rapid confirmation of disease by newborn screening allow for timely intervention.

The Norwegian NBS program is governed by a general legislation covering all specialist health services and covered by a specific regulation on the mass genetic testing of newborns. This allows genetic testing of all conditions in the NBS program by informed parental consent, but without the need for written consent and genetic counselling prior to testing [4]. In contrast to several other countries (e.g., [5]), heterozygote single carriers of the screening disorders are not reported or referred for further clinical evaluation. Carrier status is not relevant information related to the health of infants, but can potentially be relevant when the individual reaches reproductive age [6]. Carriers will be detected using both Sanger sequencing and next generation sequencing (NGS). The use of NGS methods, such as genome sequencing or gene panels, may lead to a higher risk of incidental findings. Custom-made amplicon-based gene panels, only including genes strictly related to the NBS disorders, will reduce this risk, but for other NGS methods, bioinformatics filtering is necessary to avoid revealing other diseases not part of the screening program. These are important topics to address when considering genetic analysis of newborns.

After first tier flow-injection analysis by tandem mass spectrometry (MS), immunoassays by the Genetic Screening Processor (GSP) instrument, and quantitative PCR (qPCR), the samples with abnormal results on the first tier undergo second tier biochemical analysis by MS or sequencing performed on DNA extracted from the dried blood spot (DBS) filter cards (Table 1). Molecular testing was gradually implemented into the screening algorithm when the Norwegian NBS program was expanded from two to 23 disorders in 2012 [1,2], and the Sanger method was and still is used for molecular analysis of the disorders caused by pathogenic variants in single genes, currently 13 disorders. For ten of the screening disorders, CF, T cell deficiency/SCID, methylmalonic aciduria (MMA), propionic aciduria (PA), maple syrup urine disease (MSUD), multiple acyl-CoA dehydrogenase deficiency (MADD)/Glutaric aciduria (GA2), Holocarboxylase synthetase deficiency/3-hydroxy-3-methyl-glutaryl-CoA-lyase deficiency (HCS/MCD/HMG), Carnitine-acylcarnitine translocase deficiency (CACT), Carnitine palmitoyl transferase 2 deficiency (CPT2), and long-chain-3-hydroxy acyl-CoA dehydrogenase deficiency (LCHADD)/trifunctional protein deficiency (TFP), next generation sequencing (NGS) will be performed due to these disorders’ heterogeneity with large or multiple disease genes. All molecular testing, both Sanger and NGS methods, are performed within the Norwegian screening laboratory. CH and CAH molecular testing are not part of the current screening algorithm. Excluding PKU, the introduction of biochemical and genetic second tier analyses has improved the positive predictive value (PPV) from 26% to 75% for the IEMs included in the Norwegian NBS program [1]. Moreover, the qPCR method has minimized the number of new samples and referrals from SCID screening [3] and SMA. Being allowed to avoid reporting heterozygote carriers of recessive disorders, the number of referrals in screening for CF, SCID, SMA and the IEMs are significantly reduced [1,2,3].

Sanger sequencing was first introduced by Sanger et al. in 1977 [7], and has since been the preferred method in many laboratories for detecting disease-causing variants until the introduction of NGS methods. Even though the Sanger technique has remained unchanged for approximately 30 years, developments in technology and chemistry have made it cheaper and easier to perform [8]. Most NBS programs today report pathological findings based on biochemical markers alone and do not include any second tier DBS DNA analyses [9,10]. For screening laboratories that do perform molecular analyses, Sanger sequencing has been the preferred method, especially when the suspected disease is related to one single gene or a few genes (e.g., [11,12,13,14,15]). We hereby present our current Sanger sequencing workflow in the Norwegian NBS program, including the primer sequences used for the different disorders.

**Table 1:** Current cut-off values used at the newborn screening for eliciting second and third tier methods.

Disorder	First Tier Biomarkers		Cut-Off	Units	First Tier Methods	Second Tier	Third Tier	Disease Gene(s)	Awaiting Result of DNA Test Prior to Referral
ADA-SCID	Ado	≥	3.0	µmol/L	LC-MS/MS	Sanger	-	*ADA*	YES, if >5 TREC/µL
	dAdo	≥	0.05	µmol/L					
BTD	BTD	≤	40	U/dL	Immunoassay	Sanger	-	*BTD*	YES
BKT	C5:1	≥	0.1	µmol/L	LC-MS/MS	Sanger	-	*ACAT1*	YES
	C4OH + C3DC	≥	0.5	µmol/L					
CAH	17OHP, GA ≥ 35 weeks	≥	30	nmol/L	Immunoassay	LC-MS/MS Cortisol, 21-DC, 11-DC, 17-OHP, androstenedione	-	*CYP21A2* and others	NOT RELEVANT
	17OHP, GA < 35 weeks	≥	80	nmol/L					
CACT/CPT2	(C16 + C18:1)/C2	≥	0.8		LC-MS/MS	NGS panel	-	*SLC25A20*, *CPT2*	NO
	and C14/C3	≥	0.7						
CF	IRT	≥	38	ng/mL	Immunoassay	NGS single gene	-	*CFTR*	YES
CH	TSH	≥	10	µU/mL	Immunoassay	NONE	-	Multiple, and non-genetic factors	NOT RELEVANT
CPT1	C0/(C16 + C18)	≥	40.0		LC-MS/MS	Sanger	-	*CPT1A*	NO
	(C16 + C18:1)/C2	≤	0.15						
CTD	C0	≤	6.0	µmol/L	LC-MS/MS	Sanger	-	*SLC22A5*	YES
	C3/Met	≤	0.06						
	C3 + C16	≤	2.0	µmol/L					
GA1	C5DC	≥	0.4	µmol/L	LC-MS/MS	Sanger	-	*GCDH*	NO
	C5DC/C16	≥	0.1						
GA2	C14:1/C2	≥	0.02		LC-MS/MS	NGS panel	-	*ETFA*, *ETFB*, *ETFDH*	NO
	C12	≥	0.4	µmol/L					
HCS/MCD/HMG	C5OH + C4DC	≥	1.0	µmol/L	LC-MS/MS	Sanger or NGS panel	-	*HLCS*, *HMGCL*	YES
HCY	Met	≥	40	µmol/L	LC-MS/MS	LC-MS/MS tHCY	Sanger, NGS panel	*CBS* (Sanger), *MAT1A*	YES
	Met/Phe	≥	0.7						
IVA	C5	≥	1.0	µmol/L	LC-MS/MS	LC-MS/MS C5-carnitine	Sanger or NGS panel	*IVD*	C5 > 8 µmol/L: NO C5 ≤ 8 µmol/L: YES
	C5/C0	≥	0.04	-					
LCHADD/TFP	C16OH	≥	0.1	µmol/L	LC-MS/MS	Sanger or NGS panel		*HADHA*, *HADHB*	NO
	C18OH	≥	0.1	µmol/L					
MCADD	C8	≥	0.4	µmol/L	LC-MS/MS	Sanger		*ACADM*	NO
MMA	C3	≥	4.75	µmol/L	LC-MS/MS	LC-MS/MS MMA/tHCY/MCA	NGS panel	*MUT (Sanger)* *MMAA*, *MMAB, MMACHC*, *MMADHC*, *LMBRD1*	NO
	C3/C2	≥	0.25						
MSUD	Xle	≥	250	µmol/L	LC-MS/MS	LC-MS/MS Leu, Ile, Allo-Ile, Val	NGS panel	*DBT*, *DLD*, *BCKDHA*, *BCKDHB*	NO
	Xle/Ala	≥	1.3						
	Val	≥	250	µmol/L					
PA	C3	≥	4.75	µmol/L	LC-MS/MS	LC-MS/MS MMA/tHCY/MCA	NGS panel	*PCCA*, *PCCB*	NO
	C3/C2	≥	0.25						
	C4/C3	≤	0.05						
PKU	Phe	≥	150	µmol/L	LC-MS/MS	Sanger	NGS Panel, WGS: CNVs	*PAH* (Sanger) *GCH1*, *PTS*, *QDPR*, *PCBD1*	NO
	Phe/Tyr	≥	1.5						
SCID	TREC	≤	20	TREC/µL	RT-PCR	NGS panel	WGS	Multiple SCID and T-cell deficiency genes	If <5 TREC/µL: NO 5–20 TREC/µL: YES
SMA	*SMN1*	<	2	gene copies	q-PCR	ddPCR *SMN2* copies	qPCR of one founder variant	*SMN1*, *SMN2*	YES *
TYR1	SuAc	≥	2.0	µmol/L	LC-MS/MS	Sanger		*FAH*	YES
VLCADD	C14:1	≥	0.5	µmol/L	LC-MS/MS	Sanger or NGS panel		*ACADVL* panel with GA2	C14:1 > 2µmol/L: NO C14:1 < 2µmol/L: YES
	C14:1/C2	≥	0.02						

Abbreviations: ADA-SCID: Adenosine deaminase deficiency—severe combined immunodeficiency, BTD: Biotinidase deficiency, BKT: Beta-ketothiolase deficiency, CAH: Congenital Adrenal Hyperplasia, CACT/CPT2: Carnitine-acylcarnitine translocase deficiency/Carnitine palmitoyltransferase 2 deficiency, CF: Cystic fibrosis, CH: Congenital Hypothyroidism, CPT-1A: Carnitine palmitoyl transferase 1 deficiency, CTD: Carnitine transporter deficiency, GA1: Glutaric aciduria type 1, GA2: Glutaric aciduria type 2, HCS/MCD/HMG: Holocarboxylase synthetase deficiency/3-hydroxy-3-methyl-glutaryl-CoA-lyase deficiency, HCY: Cystathionine β-synthase deficiency, IVA: Isovaleric acidaemia, LCHADD/TFP: Long-chain acyl-CoA dehydrogenase deficiency/Trifunctional protein deficiency, MCADD: Medium-chain acyl-CoA dehydrogenase deficiency, MMA: Methylmalonic academia, MSUD: Maple syrup urine disease, PA: Propionic acidaemia, PKU: Phenylketonuria, SCID: Severe combined immunodeficiency, SMA: Spinal Muscular Atrophy, TYR-1: Tyrosinemia type 1, VLCADD: Very long-chain acyl-CoA dehydrogenase deficiency. GA: gestational age, IRT: Immune reactive trypsinogen, TSH: Thyroid-stimulating hormone, TREC: T-cell receptor excision circles, SMN1: Survival motor neuron 1 gene, LC-MS/MS: Liquid chromatography coupled to tandem mass spectrometry, q-PCR: quantitative polymerase chain reaction, NGS-panels: Next generation sequencing panels, ddPCR: digital droplet polymerase chain reaction, SMN2: Survival motor neuron 2 gene, Xle: leucine/isoleucine/allo-isoleucine/hudroxyproline, tHCY: total homocysteine, C5-carnitine: isovalerylcarnitine, Leu: Leucine, Ile: Isoleucine, Allo-Ile: Allo-Isoleucine, Val: Valine, 21-DC: 21-deoxycortisol, 11-DC: 11-deoxycortisol, 17-OHP: 17α-hydroxyprogesterone. See Ljungblad et al. 2022 [16] for method details. * Awaiting results from ddPCR prior to referral.

## 2. Materials and Methods

### 2.1. First Tier Methods, Biochemistry and Biomarkers, and Algorithms for Follow-Up

The primary screening methods of all samples currently include (1) flow-injection analysis by tandem mass spectrometry (LC-MS/MS; Acquity Xevo-TQS micro, Waters, Milford, MA, USA) using the NeoBase^™^ and NeoBase^™^2 (since 2019) non-derivatized MSMS kit (Revvity, Turku, Finland) [1], (2) immunoassays by the Genetic Screening Processor instrument (GSP^®^) using the GSP Neonatal kits for biotinidase, IRT, hTSH and 17α-OH-progesterone (Revvity, Turku, Finland), and (3) qPCR of TRECs and Survival Motor Neuron gene 1 (*SMN*1) multiplexed and performed on ViiA7 and QuantStudio^™^7 Flex; (ThermoFisher Scientific Inc., Waltham, MA, USA) real-time PCR systems [3]. The cut-off for abnormal results from the first tier are presented in Table 1. Samples with abnormal biochemical values from the first or second tier, are selected for second or third tier genetic analyses, respectively. Sanger sequencing is currently the preferred method for 13 of the screening disorders (see Table 1). Sanger sequencing is also used as the third tier to confirm pathogenic variants identified both by Sanger and NGS methods. Sanger was previously used as the third tier in CF screening [2], but has been replaced by NGS since 2015. With regard to ADA-SCID, the second tier Sanger sequencing is performed after abnormal values on first tier MS (high Ado and dAdo) (Table 1). For the other SCID and T-cell deficiencies, a broad NGS based gene panel is applied as the second tier when first tier qPCR TREC testing shows abnormal low values. For all disorders confirmed by Sanger, sequencing is started as soon as the biochemical result is positive. For other SCID and T-cell deficiencies, NGS is started as soon as a TREC value is zero, or close to zero. Less urgent NGS samples are batched and run once or twice a week.

Since the IRT biomarker for CF has relatively poor specificity, NGS sequencing of all coding regions of *CFTR* are performed in as many as 5% of all incoming NBS samples. These samples are batched (44 or 92 samples per batch), and normally run within 7 days of the first screening.

### 2.2. Extraction of DNA from Guthrie Cards

DNA is extracted from dried blood spots (DBS) on Guthrie cards in a single 3.2 mm punch. The samples are washed in 150 µL Elution Solution 2 (ES2; Qiagen, Germantown, MD, USA) by incubating for 10 min on a heated shaker (1000 rpm) at 60 °C. Then the elution solution is removed, before the samples are eluted in a final volume of 100 µL ES2 and incubated for 30 min at 100 °C. This is a modified version of a previously published method [17]. One 3.2 mm punch contains on average 3 μL of blood, and the extraction method used yields approximately 30 ng of DNA from each punch.

### 2.3. PCR Amplification, Purification and Sequencing

The samples are sequenced with primers for the relevant genes related to the specific disorder using the following PCR setup: 7.5 µL of 2 × AmpliTaq Gold 360 Mastermix (ThermoFisher Scientific Inc., Waltham, MA, USA), 3 µL of 1.2 µM of mixed forward and reverse M13 tagged primers (Tables S1 and S2) and 4.5 µL of extracted DNA. The amplification is performed on a Veriti Thermal Cycler (ThermoFisher Scientific Inc., Waltham, MA, USA). The following program is used for all primer pairs: 96 °C for 5 min, followed by 40 cycles of 94 °C for 30 s, 60 °C for 45 s and 72 °C for 45 s, followed by a final extension step, 72 °C for 10 min. The samples are stored at 10 °C until the next step.

Unincorporated nucleotides and primers are digested by adding 6 µL ExoSAP-IT Express (United States Biochemical, Cleveland, OH, USA) reagent to each well with the PCR product, and run at 37 °C for 4 min followed by 80 °C for 1 min, and 10 °C until the next step.

The sequencing reaction is performed on the purified PCR products using 4 µL 1:8 diluted BigDye Terminator v.3.1 Ready Reaction Mix (ThermoFisher Scientific Inc., Waltham, MA, USA), 2 µL 5 × Sequencing Buffer (ThermoFisher Scientific Inc., Waltham, MA, USA) and 1 µL 3.2 µM M13 forward or reverse primers. The samples are run on a Veriti Thermal Cycler using the following program: 96 °C for 1 min, followed by 25 cycles of 96 °C for 10 s, 50 °C for 5 s and 60 °C for 2 min. The samples are then stored at 10 °C until cleaning of the post-PCR product.

The post-PCR products are cleaned using the BigDye XTerminator^®^ Purification Kit (ThermoFisher Scientific Inc., Waltham, MA, USA). A mix of 10 µL XTerminator and 45 µL SAM solution is added to each 10 µL BigDye product, followed by shaking at 2000 rpm for 20 min on a Thermomixer C (Eppendorf, Hamburg, Germany). The samples are centrifuged for 1 min at 1000 rpm before being placed in a 3500 DX Series Genetic Analyzer (ThermoFisher Scientific Inc., Waltham, MA, USA) with POP-7 polymer (ThermoFisher Scientific Inc., Waltham, MA, USA) and a 50 cm array length for sequencing.

### 2.4. Analysis, Classification and Reporting

The Sanger sequences are analyzed using Variant Reporter^™^ Software v3.0 (ThermoFisher Scientific Inc., Waltham, MA, USA) with the correct NCBI Reference Gene Sequence aligned to each primer pair. Novel gene variants are evaluated using Alamut Visual (Sophia Genetics, Lausanne, Switzerland), ClinVar [18], GnomAD [19], HGMD [20], and a search for relevant publications. ACMG criteria are used for the classification of variants. All samples, homozygous or compound heterozygous, for two pathogenic variants in autosomal recessive disorders are reported as screening positive (see Table 2 for definitions).

Carriers for autosomal recessive and SCID X-linked recessive disorders are defined as screening negative. Exceptions are screening samples with extreme values on the first tier biochemical or qPCR analyses. These samples are reported as screening positive regardless of the results from the sequencing, or prior to completion of the molecular testing for disorders where time is critical to prevent detrimental outcome (see Table 1). For all samples where two pathogenic or likely pathogenic variants in the same gene are identified, either by Sanger or NGS, DNA is extracted from a new punch from the same original filter card. The new DNA extract is sequenced using Sanger with selected relevant primers covering the nucleotide positions containing the identified variant.

## 3. Results

Sanger sequencing was introduced as a second tier test in the Norwegian newborn screening program in 2012, starting with the eight following autosomal recessive disorders; CACT, CPT1, CPT2, IVA, LCHADD/TFP, MCADD, PKU and VLCADD (See Table 3 for abbreviations). The application of this sequencing method has gradually expanded to include 16 disorders (three of these are often run on NGS due to complexity with other diseases) in the program in 2022 [1]. A total of 668 samples have been sequenced using Sanger sequencing from 1 March 2012 to 1 April 2022. In 191/668 (28.6%) samples, two pathogenic/likely pathogenic variants were identified, and these samples were reported as screening positive. Samples with a clear biochemical profile on the first tier, and one pathogenic or likely pathogenic variant in combination with a VUS evaluated to have possible protein changing effects or possible splicing defects, were also reported as screening positive. In 147 samples, the infants were carriers of one pathogenic variant of an autosomal recessive disorder and in the remaining 330 samples, no pathogenic variants were detected. Heterozygote carriers and samples without any pathogenic variants were not reported (Table 3). Exceptions to this approach are samples with clear biochemical results. These will be reported as screening positive regardless of the sequencing results (see Table 1). As a result of this, no false negatives have been reported as a result of incomplete Sanger sequencing, so far. Sanger sequencing was used as a third tier in the CF screening from 2012 to 2015 but has later been replaced by NGS. The *CFTR* Sanger sequencing results have been published previously [2].

When sequencing was introduced in 2012, samples were manually requested for genetics, after individual medical evaluation of the first tier biochemical values or biomarker levels. Fixed cut-off values for automatic requisition of Sanger sequencing were established in early 2018. The threshold values were set based on experience with the biomarker levels and biochemical patterns of the various screening disorders, as well as the sequencing results from 373 samples. There have been some adjustments over time to these cut-off values used for initiating sequencing, as well as inclusion of new biomarkers in the second tier for certain disorders, which have led to a reduction in the number of samples sequenced, with no apparent increase in the number of false negatives.

As an example, the cut-off values for biotinidase (BTD) activity prompting second tier analysis, was lowered from 60 U/dL to 40 U/dL in 2018. The algorithm was changed since a large portion of the samples sequenced with enzyme levels above 40 U/dL were either mutation negative, heterozygote carriers, or had variants earlier classified as pathogenic, but by previous referrals demonstrated to have sufficient enzymatic residual activity. An example of a *BTD* variant no longer reported as homozygous or compound heterozygous is p.Asp444His. This variant is classified in ClinVar as pathogenic (ACMG score 5), but clearly related to a very mild phenotype. So far no false negatives as a result of lowering the cut-off value of biotinidase have been reported.

Nationwide SCID screening was implemented in Norway in January 2018 [3]. In 2019, the transition to a new version MS (NeoBase^TM^2) kit, introduced the option to measure adenosine, which is a marker of adenosine-deaminase deficiency and ADA-SCID. At first, the cut-off value for sequencing the *ADA* gene was set too low (Ado > 1.61 μmol/L) causing a large number of samples to be sequenced, without finding any pathogenic variants. At the end of 2019, the cut-off value was increased to Ado > 3 μmol/L. During 2020, only four samples were *ADA*-sequenced, all wild-type, supporting the decision to increase the cut-off value. In 2021, the cut-off for an additional marker (dAdo ≥ 0.05 µM) for ADA deficiency was included in the screening. Since 2021, only one true positive ADA-SCIDs has been detected, with Ado and dAdo significantly above the cut-off. Individuals with ADA-SCID usually, albeit not invariably, also have low TREC values on first tier qPCR.

We have experienced a single false positive screening result due to Sanger sequencing of DBS. This was a sample with abnormal results on the first tier (C0:5.2, C3 + C16:1.9, C3/Met:0.03) where we identified and reported a homozygous pathogenic *SLC22A5* variant (NM_003060.3(*SLC22A5*):c.506G>A(;)506G>A, p.(Arg169Gln) hom). Later, based on parental testing, it was concluded that the child was only heterozygous for the variant, and the child did not have carnitine transporter deficiency. A single nucleotide polymorphism (SNP) in the primer binding site had caused allele dropout of the wild-type allele. The primer sequences have since been updated to avoid covering common SNPs.

## 4. Discussion

As of 2023, many NBS laboratories rely solely on biochemical results [22]. It has previously been shown that combining the use of biochemical markers with genetics increases the PPV, and significantly reduces the number of false positives (e.g., [1]) and false negatives (e.g., [23]) in newborn screening for IEMs, CF and SCID [1,2,3]. In the Norwegian NBS, the PPV for metabolic diseases has increased from 26% the first year of expanded screening, to >70% in 2021. [1]. For Biotinidase deficiency (BTD), Carnitine transporter defect (CTD), and very long-chain acyl-CoA dehydrogenase deficiency (VLCADD), Sanger has decreased the number of positively reported samples drastically (Table 3), compared to relying on biochemistry alone. However, for some of the newborn screening disorders the biochemical markers still remain the sole parameters for both screening and confirmation (Table 1).

There are benefits and disadvantages with all sequencing methods, and choosing which one to use in different situations is dependent on several factors. For the sequencing of single genes in single samples, Sanger sequencing is ideal. It is rapid, with results available within one working day. The hands-on time is less than the time spent preparing NGS libraries, and the analysis of the results is less time-consuming. In addition, Sanger sequencing of a single or a few genes is more cost efficient than NGS analysis (e.g., [11,12,13,14,15,24,25,26]). However, Sanger sequencing is only suitable for small or a few genes, and is unable to detect large insertions and deletions. Therefore NGS technologies, such as whole genome sequencing, amplicon based, targeted/broad, and custom-made gene panels are useful and complement Sanger sequencing. NGS technology is already established in the Norwegian NBS program, and it is currently being introduced in several other NBS programs in Europe (e.g., [3,27,28]).

Since the introduction of Sanger and genetics as a second tier in 2012, a total of 191 screening positive samples have led to referral in Norway. All true screening positive patients receive life-long follow-up, as part of the NBS program, and most patients are admitted to Oslo University Hospital or receive follow-up in close collaboration with the local hospital. Due to prompt DBS DNA Sanger sequencing, in many cases, the diagnosis is genetically confirmed before the newborn is referred. This reduces time in diagnostic uncertainty for the family and severe complications for the infant, since intervention can be initiated prior to any signs of symptoms [29]. In a few instances where a clinical evaluation of the biochemical results has correctly identified a positive case, Sanger sequencing has failed to confirm the molecular diagnosis. There are examples of patients with biochemical values consistent with PKU, where Sanger sequencing only detected a single heterozygous pathogenic variant. The other pathogenic *PAH* variant was a structural variant, which was later detected by another method. When no *PAH* variants are detected in a child with hyperphenylalaninemia, the genes for the various tetrahydropterin BH4 deficiencies are tested, such as *GCH1*, *PTS*, *QDPR*, and *PCBD1* (Table 1). Additional methods such as whole genome sequencing or long read sequencing may aid us further by unravelling structural variants and deep intronic variants which confirm the molecular cause of the diagnosis. Targeted Sanger sequencing can then be used to confirm the pathogenic variants found using these methods. In all cases it is important to note that sequencing results are used as timely information for decision support and do not override a complete clinical evaluation of the available results at the time of reporting.

Compared to NBS decision-making, based solely on abnormal first tier biochemical results, almost 500 families have been spared a false positive NBS referral due to second tier genetic testing since 2012. Although follow-up testing at the hospital would in most cases rapidly conclude that the child was healthy, anxiety and stress in a sensitive time with a newborn have been avoided in these cases.

## 5. Conclusions

The Norwegian NBS program has implemented genetic testing from the NBS filter card as a second or third tier analysis for the majority of the included screening disorders. Here, we have described our use of Sanger sequencing as an easy, reliable and quick tool to support the NBS decision making. The method is ideal for identifying the genetic cause of disorder caused by single genes and a useful tool to confirm variants identified by NGS based gene panels. Genetic testing by Sanger sequencing has contributed to a substantial increase in PPV in the Norwegian NBS.

## Figures and Tables

**Table 2:** Definitions of the terminology recommended in newborn screening.

Terminology	Definition
Referral	A newborn that is immediately, after abnormal screening, referred to and clinically evaluated by a pediatrician with follow-up diagnostics and therapeutic intervention.
Screening positive	A final, reportable result for a specific disorder or group of disorders based on the newborn screening test result(s) and screening algorithm, indicating high risk of the disorder(s) and the need for clinical evaluation, confirmatory tests, treatment and follow-up. The terms “abnormal value” and “urgent abnormal value” are recommended to be used for this category [21].
Screening negative	A final, reportable result for a specific disorder or group of disorders based on the newborn screening test result(s) and screening algorithm, indicating low risk of the screening disorder(s), no need for confirmatory testing, intervention or additional follow-up. According to Blom et al. [21] the term “normal value” is recommended to be used for this category in SCID screening, but it does not reflect the whole complexity of the screening negatives.
Screening carrier	A newborn that is heterozygous for one pathogenic variant identified by sequencing, and the variant is located in a gene for an autosomal recessive disorder related to the abnormal first tier test results.

**Table 3:** The number of samples that were Sanger sequenced from 2012–2021 for each disorder, number of screening positive, screening negative and carriers found.

Starting Year	Screening Disorder	Sanger Sequenced	Screening Negative	Screening Carrier	Screening Positive
2012	CACT	19	18	0	1
2012	CPT-1A	11	7	1	3
2012	CPT2	19	15	0	4
2012	IVA	7	2	0	5
2012	LCHADD	4	1	0	3
2012	MCADD	62	9	33	20
2012	PKU	99	3	8	87 + 1 PTPS
2012	VLCADD	100	61	30	9
2013	BKT	11	10	1	0
2013	BTD	70	17	25	28
2013	CTD	133	84	41	8
2013	GA1	13	6	0	7
2013	TYR-1	21	10	2	9
2014	HCY	45	38	4	3
2014	TFP	4	0	1	3
2019	ADA-SCID *	50	48	1	1

* For the other SCID and T-cell deficiencies a broad NGS based gene panel is used as the second tier when first tier qPCR TREC testing shows abnormal low values [3]. Abbreviations: ADA-SCID: Adenosine deaminase severe combined immunodeficiency, BKT: Beta-ketothiolase deficiency, BTD: Biotinidase deficiency, CTD: Carnitine transporter deficiency, CACT: Carnitine-acylcarnitine translocase deficiency, CPT-1A: Carnitine palmitoyl transferase 1 deficiency, CPT-2: Carnitine palmitoyl transferase 2 deficiency, GA1: Glutaric aciduria type 1, HCY: Cystathionine β-synthase deficiency, IVA: Isovaleric acidemia, LCHADD: Long-chain acyl-CoA dehydrogenase deficiency, TFP: Trifunctional protein deficiency, MCADD: Medium-chain acyl-CoA dehydrogenase deficiency, PKU: Phenylketonuria, TYR-1: Tyrosinemia type 1, VLCADD: Very long -chain acyl-CoA dehydrogenase deficiency. Some samples were sequenced using both Sanger and NGS panel sequencing, and the results are only counted once. *CACT* and *CPT2* are now mainly run together on an NGS panel. The same is true for VLCADD and GA2. Here, several genes (*ACADVL*, *ETFA*, *ETFB* and *ETFDH*) have overlapping biochemical results and can cause disease.

## Data Availability

The data presented in this study are available in Table 3 and Supplementary Tables S1 and S2.

## Supplementary Materials

The following supporting information can be downloaded at: https://www.mdpi.com/article/10.3390/ijns9040067/s1, Table S1: Primer sequences used for the conditions mainly sequenced using Sanger sequencing, Table S2: Primer sequences used for Sanger sequencing of the conditions mainly sequenced using panel sequencing. Reference [30] is cited in the Supplementary Materials.
