# Supplementary material for: Newborn Genetic Screening—Still a Role for Sanger Sequencing in the Era of NGS
Source: Int J Neonatal Screen. 2023 Dec 7;9(4):67. doi: 10.3390/ijns9040067 (PMC10743540; doi:10.3390/ijns9040067)
Supplement: Supplementary file 1 [file IJNS-09-00067-s001.zip › IJNS-2642653-supplementary.pdf]

Table S1. Overview of the different primers used for Sanger Sequencing.

Disease (Gene)	Transcript	Covering	Forward primer	Reverse primer	Primer designer
BKT (ACAT)	NM_000019.3	Exon 1	TGTGCTGGGTAGGCCTAAGGG	ACCCGTCGCGTGACTGTCCT	Variant_Seqr
	NM_000019.3	Exon 2	ATGTGCCACAATGCTGGGCT	CCTGCTTTGACTTTCACATGTATCCAA	Variant_Seqr
	NM_000019.3	Exon 3	CCTCAAACCTCCTGTAGAGAACTGGG	CAAGGGAAAGCAGAAATGTAGGCA	Variant_Seqr
	NM_000019.3	Exon 4	GCAGGTCAGTAGTTACTTGGCTTT	CCGGCCCCTTTTACACATTTTAAGT	Variant_Seqr
	NM_000019.3	Exon 5	TCTGCAGTTGTGTTTGAGTATCGGTT	TCAAATGGACTTAGCAAATCCAGACA	Variant_Seqr
	NM_000019.3	Exon 6	CATCAGGGTGAAGTGGTAAAGAGGG	GCAGTTGCCAGCTAAGCGTCAG	Variant_Seqr
	NM_000019.3	Exon 7	CCACCACCTCCGGCCTAAGA	TTCTTGGAGGCACTCCCGGT	Variant_Seqr
	NM_000019.3	Exon 8	GGGATGGTTTAAGTGAAGCAGGG	TCAATGCCAATGAAGACCCTATCA	Variant_Seqr
	NM_000019.3	Exon 9	AAACATTTAGCAGCCCAGGCAA	AGGCACATGCCACCATGCTC	Variant_Seqr
	NM_000019.3	Exon 10	TGGTGCCAATGTTCCTAGAGGC	CCAAGCTATCTCTCTGCTTTGGCA	Variant_Seqr
	NM_000019.3	Exon 11	TTCCATCTGAAACCAAGAACTTCCA	TGCCTTATGAAAGATGGGTAGGAAGG	Variant_Seqr
	NM_000019.3	Exon 12	CCCTTCCTGAAACAAGTCCTCCA	GCTGACCCACAGTAGTCACACTGATT	Variant_Seqr
ADA (ADA)	NM_000022.2	Exon 1	GCCTAAGCCGAAGGAAGAACTCG	CCGTTAAGAAGAGCGTGGC	In house design
	NM_000022.2	Exon 2	GCTTGATTCCCACAGGGAGAC	AACATTAAGCTCTGAAAGGTCCTTCG	Primer Designer
	NM_000022.2	Exon 3	CTCCCATATTCTGCCATTTCTCTGT	TGAGAGACGCCCATGTCTCCT	Primer Designer
	NM_000022.2	Exon 4	CGGCAAAGGGAGGATGGGAAC	TTGCCCAGTGTCTGGGATCTTAT	Primer Designer
	NM_000022.2	Exon 5	GGAGATCAGGTACAGCCCCACT	GTGAACATCATGGCAGGCCCAA	Primer Designer
	NM_000022.2	Exon 6	TAAGACCCAACAAAGACACACTTCA	GACACTGAGCCCTGTTTTGTGCT	Primer Designer
	NM_000022.2	Exon 7 and 8	GCCCTGCTTTCTGCCTCTCTAT	AGTGACAGCTACCACTTAGATAAGG	Primer Designer
	NM_000022.2	Exon 8 and 9	GATGCCCAATCCCTAAAGTTTCTTC	AGTAGTAAAAGAGGTGAGGGCCTG	Primer Designer
	NM_000022.2	Exon 10	CCTCTCTCCAAAGATTCCAGGC	GGCTGCCATTCTGCCTGGTT	Primer Designer
	NM_000022.2	Exon 11	CCAGAAACCAGGGAGTCATCAC	GGTGTCTCTGGCCCCCTACAT	Primer Designer
	NM_000022.2	Exon 12	TGTAAAAATGTTGCTCAGCCCCA	GCCCCAGTGTGGCTTTCTGTG	Primer Designer
BTD (BTD)	NM_000060.3	Exon 1	CGTCCACTTCCCGGGAGAG	CAGCTCCCGGCAGCCTTTG	Variant_Seqr
	NM_000060.3	Exon 2	CAAACTCTTTGAGCCGCAGTATCAC	CCTGGATGCTTTTTCAGAGGGTGG	Variant_Seqr
	NM_000060.3	Exon 3	GCCTCTGGTTCCTGCTACAGAGTAAC	GGATTTTGGGGATGTTAAGGTCTCCT	Variant_Seqr
	NM_000060.3	Start exon 4 (to AA280)	CACCGTGTTAGCCAGGGTG	CTGCCAAGAGTGGGAGCTGG	Variant_Seqr
	NM_000060.3	Exon 4 (AA253 -> AA442)	CTGCCATCAGAGTCCTCAGAGAC	ACCCCCAGGGCATACAGCTC	Variant_Seqr
	NM_000060.3	Exon 4 (AA417 -> end)	CGTCTGTTCCAATGGCCTCTG	CTTGTAGCCTGTGGAAGTGC	Variant_Seqr

CPT1A (CPT1A)	NM_001876.4	Exon 2	AACGCATGCCAAGTCCCTCC	TGCCAGCTGTGGTGATGGAA	Variant_Seqr
	NM_001876.4	Exon 3	ATTGGCCAGGCTAAGGGCAC	TCTCAGTGCACGCACGCTGT	Variant_Seqr
	NM_001876.4	Exon 4	GCCATTCAAGGTTGAGGCCC	CCCTCTGACTTTCCCGGCTG	Variant_Seqr
	NM_001876.4	Exon 5	CTTGAGCCAAATGGCGGTGA	TATTGGCATGGAGGGCAGGG	Variant_Seqr
	NM_001876.4	Exon 6	GCTGGTGTACCTGCACTCAGGG	CCTAAGGCACAAGTGAGGCAAACA	Variant_Seqr
	NM_001876.4	Exon 7	GTTCTCCGGCCTGGAAGCAG	TCCCTGTTCTGGAATGGCCC	Variant_Seqr
	NM_001876.4	Exon 8	GATGATCCGCCTGCTTTGGC	TGTTACGTGAGGGTATTTGGTGCAA	Variant_Seqr
	NM_001876.4	Exon 9	GCATAGGGATGGAAAGTGCCG	TCAAACGAAGCAATTCGCACA	Variant_Seqr
	NM_001876.4	Exon 10	TCTTGAGACCTGCCAAGCCG	GCCTGGAGAGACGCAGAGCA	Variant_Seqr
	NM_001876.4	Exon 11	GGTGAGTGTCAGGCACACAGGG	AGCCTGCAATCTGGCCGTTC	Variant_Seqr
	NM_001876.4	Exon 12	AAATTGACTGTGGGGTTTGGA	TTGTGTCTGGGACAGGGAAG	Variant_Seqr
	NM_001876.4	Exon 13	CCGAGGCAACAGGAGCTGTG	GCCCTTAGCCCTCGTCTGTCC	Variant_Seqr
	NM_001876.4	Exon 14	TCTCCTATGCCGGGTTTCGG	CCTGCTCATGTCAGACCCGC	Variant_Seqr
	NM_001876.4	Exon 15 (Missing AA 623->625)	TGGCACATTACCGTCTGGGC	TGCTGCCACGTCACTACCCA	Variant_Seqr
	NM_001876.4	Exon 16	GCAGTACTAAACGCGCTGCTCA	CAAACTTCATTCTGGAAAGGCTGTCA	Variant_Seqr
	NM_001876.4	Exon 17	TACGGCGGTAGAACCGCAAG	CAGATACGCTGCCCAAGCCA	Variant_Seqr
	NM_001876.4	Exon 18	TCCCGAAATCAAGTAAAAGCAGGC	ATGGATGTCCTTGGCAGTCTT	Variant_Seqr
	NM_001876.4	Exon 19	AAATCCAAGCCGATGCGGAG	CGACAACACACCCGCACAAA	Variant_Seqr
CTD (SLC22A5)	NM_003060.4	Exon 1 (start-> AA125)	CCTCCGCGGACGGTCTTG	AGGTAGACGTCCTGACTGAACT	Primer designer
	NM_003060.4	Exon 1 (AA88-> end Exon 1)	ACCATCGCCAACTTCTCGG	CAAGTCCTGGCTCTGCTACTTACC	Primer Designer
	NM_003060.4	Exon 2	TGGCAGGATGTTCTGACTTC	ACTACCAACTGAAATCAAGGG	Primer Designer
	NM_003060.4	Exon 3	GGATCTTGAGAAAGCCCCACTT	GTACTAAGACCAAGGAGCCACAG	Primer Designer
	NM_003060.4	Exon 4	ACTGCTAACTCGACCTCCC	AATCATCCTGCCAGTGGGCA	[30]
	NM_003060.4	Exon 5	CTATGGCTGTGCTCTACCTG	CTCAAATCACGGTCAGTCTG	[30]
	NM_003060.4	Exon 6	CTGAGTCTCTGACCACCTC	TTGTCTGGAAGCCTCAGGCA	[30]
	NM_003060.4	Exon 7	TGGGAAAGATGTGGATACTGC	GAGACAGCCTGGTAGACAG	[30]
	NM_003060.4	Exon 8	TCAATAGCTGCATGCCATGG	GCTCACATTCAAGCCAGTTAG	[30]
	NM_003060.4	Exon 9	GCATAAAGGGGTAGATGAGAG	TATTGTGAGGGGCTCCTGAG	[30]
	NM_003060.4	Exon 10	AGGATTCTCTTCCCAGGGAAG	GCAAGACAGTCTTTCCTCTTCAG	[30]
GA1 (GCDH)	NM_000159.3	Exon 1, 2 and 3	AAAGGCAGAGCTGCGGAGGG	CCTCTGACCCTGGCCACGAC	Variant_Seqr
	NM_000159.3	Exon 4 and 5	GGGTAGGGCTGATGAGGGTCC	ATACAGAGGGTTCTGCAGTGTGT	Primer Designer
	NM_000159.3	Exon 6	CAGCTAGGGCCAGCTTGGTG	TCCAGGTGAAGCCCAGGGTC	Variant_Seqr
	NM_000159.3	Exon 7 and 8	GGGATGTATCAGGGACCAGGCA	CACGGAGCCTGCCTGACAAA	Variant_Seqr

	NM_000159.3	Exon 9	TTTCCCTGCTTCAGAGTTGGTT	GGAGTCACCCATGGTATAGAGAGT	Primer Designer
	NM_000159.3	Exon 10	CCCACCAGGCCTGAGTTCCT	CATCCCGATGCTCCAAGCAA	Variant_Seqr
	NM_000159.3	Exon 11	CCTGAACCTTCTGCTGTCCCTC	TCCCGAGCATGTGGGAGAAA	Variant_Seqr
	NM_000159.3	Exon 12	TCAGCATTCACCATCTCTGTTGGTC	CCTCACTCTGCTCCAACACCCA	Variant_Seqr
HCY (CBS)	NM_000071.2	Exon 3	TCATCTACACGGCATGGCCC	TCTGAGGGAGCAGAGCACGC	Variant_Seqr
	NM_000071.2	Exon 4	CACACCCTCGCTGCTTCCAC	CCAGTTCTTCGAGTGTTGATTTGTGT	Variant_Seqr
	NM_000071.2	Exon 5	GTGCAAGATGCCAGGGAGCA	TGCCCAGGTGCAGGTGTAGG	Variant_Seqr
	NM_000071.2	Exon 6 and 7	GAATGCCTGTCTCCAGGCCC	GCAGAAGCAGCCATGTTGGG	Variant_Seqr
	NM_000071.2	Exon 7 and 8	CAGCCATGCCCTGTGTTTGC	AGGTGCAGGCCACCGCTTT	Variant_Seqr
	NM_000071.2	Exon 9	AGCTCCTTCGGGTCACGAGC	CAGACCAAGGGCAGCAACGA	Variant_Seqr
	NM_000071.2	Exon 10	CTTGGTGAGGTCAGGCCACG	TTGGAGCCTGGGTTCTTGGG	Variant_Seqr
	NM_000071.2	Exon 11	GTGGCTGACTGAGGGTGCG	GAGCAGGGAAAACGTGTGAAAATT	Primer Designer
	NM_000071.2	Exon 12	CGGTTCTCAGGTGAGGCGTG	GCTCAGGGCCAGGAGGTTTG	Variant_Seqr
	NM_000071.2	Exon 13	TGACAGGCCCAGCTCCTGAA	TGAGCGATGGCTGTGTGCAT	Variant_Seqr
	NM_000071.2	Exon 14	ATGGCTGCCCTGTCCAGTGA	CCTGAGCGACAGGTGGATGC	Variant_Seqr
	NM_000071.2	Exon 15	CGCGCACCCGCTTGA	AGAGGACTTCCATGTGTGGC	Variant_Seqr
	NM_000071.2	Exon 16	CTGCATGCTGGACAAAGGGC	GGCTTTGCAGGACCCACCAT	Variant_Seqr
	NM_000071.2	Exon 17	GAGACGGATGCTCTGTGCCG	CTGGGCTCCAGCCACCCT	Variant_Seqr
IVA (IVD)	NM_002225.3	Exon 1	CGCAGTTAGGGGCTGCTATT	CTTCACTCCACAGACGTGAG	Variant_Seqr
	NM_002225.3	Exon 2 and 3	TCTGAGGAGGCATAAACTACTGAGGC	CAATCTTTCCCTTCCCTTCCTGC	Variant_Seqr
	NM_002225.3	Exon 4	GCAGGTCTGTGGCATCAGCTT	TGTTCAATGCAAGAGTTCTGGTCC	Variant_Seqr
	NM_002225.3	Exon 5	TCAGCCTTGTAGCCATTGGGC	CCACAATGAAGGCTGTGATGCC	Variant_Seqr
	NM_002225.3	Exon 6	CTGGCCATGAGTGAGCCCAA	TGACCCATCTGCCCTGGACTT	Variant_Seqr
	NM_002225.3	Exon 7	GGCGTTAGCACCGGAAGGTC	TGCGGAGTGTGTGATGCCAG	Variant_Seqr
	NM_002225.3	Exon 8	GCCTTTGTGGTTGGCCTCGT	AGAGCAGGGCCAAGAGGCAG	Variant_Seqr
	NM_002225.3	Exon 9	ACGACACCTGGGCTGTCACG	CCAGCAGCTTCTTCGGACCA	Variant_Seqr
	NM_002225.3	Exon 10 and 11	CCATCATCCTGAGTAAATCCCATCT	GAAACTGCTTTCTGAAGCAGCCA	Variant_Seqr
	NM_002225.3	Exon 12	TGTGGCCTGTTTCTATATACTTGGCAT	GCAAGGGCACAGAGTCACCAA	Primer Designer
LCHADD (HADHA)	NM_000182.4	Exon 1	ACAGAGGGCTGCGTCTCCG	CCGTCTCACCTTGGCTGACG	Primer Designer
	NM_000182.4	Exon 2 and 3	CATGTAAGATTACTGCCAGATTGGT	TCTGACCTAACAACTGAAATGGAAAA	Primer Designer
	NM_000182.4	Exon 4	TGTGTCACTGTGCCCAGCACTT	TGGGCCAAAGCTGCCTGAAT	Variant_Seqr
	NM_000182.4	Exon 5	CAGCTGATGAATGCCACCAATG	TGCTGCTATTGTGCTATTTGAAGCAA	Variant_Seqr
	NM_000182.4	Exon 6	TCGCTTGAACCTGGGAGGAGA	AATGCCAGCCTAAGGAATTTCAGTTT	Variant_Seqr
	NM_000182.4	Exon 7	TGAGCTTCTCTCTGTGCTTCCTGTG	CCCTATATTCTCATCCTTGCTTGCC	Variant_Seqr
	NM_000182.4	Exon 8	TGTTAAAGCTGGGCCTTGGGT	TTGCCACCTACCAGTCTCCAGTC	Variant_Seqr
	NM_000182.4	Exon 9	CAGCTTGCTATGCCCTTCCCT	ACCCAAGGCCCAGCTTTAACA	Variant_Seqr
	NM_000182.4	Exon 10	GGGTATTCTTAGCATCCACTTGCTCA	TCAGTCGAGAGCAGCCAGATCC	Variant_Seqr

	NM_000182.4	Exon 11	GGGCTCAAATGTGCTCAGGAAA	CAGACTTCTGCAGCACTGAACCC	Variant_Seqr
	NM_000182.4	Exon 12	TCCGCAGGCCTGATACGAAA	TGTGCCTGCATTAGTTGGTTTATTGA	Variant_Seqr
	NM_000182.4	Exon 13	GCAATCATGAGTTTCCTATAGCTCCTT	TTGGGCATTTGGAGGCATTCTA	
	NM_000182.4	Exon 14	TTAGGAACCACCCGCATCCA	GAGGAGGTGACACCCATGCTG	Variant_Seqr
	NM_000182.4	Exon 15	CTGGGATGGCTGGGAGAACC	CCCATGGAACCAAACCACTCA	Variant_Seqr
	NM_000182.4	Exon 16	GCAGAGAGAGGGCACCAGGG	GCAGGGTTCTCCCAGCCATC	Variant_Seqr
	NM_000182.4	Exon 17	CGCCAGTGATAAGGGCTCTGTG	GTCCTGGGCCTGGTCAGGTG	Variant_Seqr
	NM_000182.4	Exon 18	TGGGAAGCTTTGGGCTGTCA	TCAGGAATTCAGAAGAGGCCAGA	Variant_Seqr
	NM_000182.4	Exon 19	CAGCTAGCAGCTGGCATGGG	TCTCTGAGGGCCAGCACCAA	Variant_Seqr
	NM_000182.4	Exon 20	GGCGTTTAAACCAGAAGGGCA	AGCCCGTGTTGCGTCTCCTT	Variant_Seqr
TFP (HADHB)	NM_000183.2	Exon 2 and 3	GCCATGGATGAGGTTATCAGAGTTCA	TCCAGCAGGTTCAAATCCCAGA	Variant_Seqr
	NM_000183.2	Exon 4	TAGCTGGGCATGATGGCAGG	TGTTGGAAGCAAGCTATCTGGACC	Variant_Seqr
	NM_000183.2	Exon 5	TGAAATGATGGACTGCCTTGGA	GGCTGATCAATCGAGTCCTGTGG	Variant_Seqr
	NM_000183.2	Exon 6	TGAGAAGGTGCCAAATGCTTGTC	CAAGAGACAATGTCCTAAACCAGCTCA	Variant_Seqr
	NM_000183.2	Exon 7	TCTGCACTAGAAACCAGCCAGCA	GCGAGATCCCGTCTCCACAA	Variant_Seqr
	NM_000183.2	Exon 8	CGTCCATATGGCAGGAATGAAAC	AGAGGATCTCTAAAGAGGGCTCACAAA	Variant_Seqr
	NM_000183.2	Exon 9	TGGCAGTGTGGACTCTGCTATGC	GGCTGAATACTTTGGAAACAGAACCA	Variant_Seqr
	NM_000183.2	Exon 10	TAAGGCTTGTGGTTCCATAGAGTTAAAAA	CCCTCTATTCATAACACACACAAAAAGTT	Primer Designer
	NM_000183.2	Exon 11 and 12	GCCTTCTCTACCCTCATCCTCCA	CACATAAACCAATTATGAACTCAGGGC	Variant_Seqr
	NM_000183.2	Exon 13	TGTTCCGTTCCCTCACAAGGC	CCCTCCCAGCAGTGTGAATCAG	Variant_Seqr
	NM_000183.2	Exon 14	GAAAGCGTAGAGGAACATGAATAACGA	AGGAGGCAATCCAACCTGGC	Variant_Seqr
	NM_000183.2	Exon 15	TGTTCCTTGCATTCTGACATTAGATGA	TGGAGGGTCCTATGACACCACTG	Variant_Seqr
MCAD (ACADM)	NM_000016.4	Exon 1	AGCCCAACCGCCTCTTCCC	CCCGTAAGCAACGTGGAAACG	
	NM_000016.4	Exon 2	CAAACCAGTTGCTGTACTCACT	TTGTGGCATTCAGATATGTAAGTTGA	Primer Designer
	NM_000016.4	Exon 3 and 4	ATGCACTGTAGTCTGAGTTTCTGAT	GCAGGAAGACAAAGGTAGAAAGTC	Primer Designer
	NM_000016.4	Exon 5	TGTGCCAGCCAGAACACATGTAGA	GTGGCAGCAAACCATCCCAA	Variant_Seqr
	NM_000016.4	Exon 6	GCAAGGTATAGGCCAGTTCTTTGGA	TTGGCATCCTTGACACATCATTG	Variant_Seqr
	NM_000016.4	Exon 7	ACAATCCTGTTTCCAAACAGTCAAA	CAGGCACTATACCAAAGGTTCTC	Primer Designer
	NM_000016.4	Exon 8	TGCGGGTAACTGAAACCACAGA	AGCCTTCTCCACAGGTTGGTACTACTC	Variant_Seqr
	NM_000016.4	Exon 9	CAGGCATTGCAGACTACAGTTTGTTG	AGCTGGGCAGAGTGGTGTGC	Variant_Seqr
	NM_000016.4	Exon 10	TTACGTGCCTATTCCCTGTTCCC	TGCATAGCACAGTGTCAGGTTCAGA	Variant_Seqr
	NM_000016.4	Exon 11	TGCTTGAGGCCAGGAGTTGC	TGAATATTCTCTCTCCTTGCAAACCAA	Variant_Seqr
	NM_000016.4	Exon 12	TCAGGTTTTGGAATCTGTAGAGGC	CTTTCTAAGCCCACTTTTCTGTCA	Variant_Seqr
PKU (PAH)	NM_000277.1	Exon 1	GCAATGGTCATTCATTCCTGTGAA	CAGCGAAGTGGTGCCTCCTG	Variant_Seqr
	NM_000277.1	Exon 2	TCATCACAAAGTTCCTGTTGAAACTGA	TCATTGATCATTTAATTGCCCTGGA	Variant_Seqr
	NM_000277.1	Exon 3	CCAGGCACTTGCCTAGGTCCC	TCTGCATCTTTGGCCTGCGT	Variant_Seqr
	NM_000277.1	Exon 4	TCCCAGCCCTCGTGTAAATAGGA	GACGGGTGGGAGGGAGATGA	Variant_Seqr

	NM_000277.1	Exon 5	AGCTTGGGAGAGAGCAGGGC	GCAAGGAGGCTCATGCTAAATCA	Variant_Seqr
	NM_000277.1	Exon 6	TGGAGGGAAGGCAGAGCACA	CCAAATCACCCTTTCATGTGGG	Variant_Seqr
	NM_000277.1	Exon 7	GCTTGGCTGCCACTTGTTCC	TCCACTACCTAAAGGTCTCCTAGTGCC	Variant_Seqr
	NM_000277.1	Exon 8	TCCTGCATGGGAATCAGGGA	TGTGCCCTACCCTGCACCTG	Variant_Seqr
	NM_000277.1	Exon 9	TTGTGCAAATGTAACCCACCACA	TCAGGGTCTATGTGGGCTGTTCTG	Variant_Seqr
	NM_000277.1	Exon 10	GCCTTGGAATCAGGAGGCCC	TCCATCTGGGATTCCAGCCC	Variant_Seqr
	NM_000277.1	Exon 11	GCAAAGGTCAGCATGCCCAA	TGGTTGGCTCCCACGTCATC	Variant_Seqr
	NM_000277.1	Exon 12	GGATGGAGAGGCGAGGGAGA	CCTCAGCCCTGGCTGTTGAA	Variant_Seqr
	NM_000277.1	Exon 13	GCTTGAATGAAGCAGGTCCCAA	TCCCTGGGAAGTGACCTATGGA	Variant_Seqr
TYR (FAH)	NM_000137.2	Exon 1	GGGTCCCTGCTGTGTCACCC	TGGGCGAGAGAAACACACCG	Variant_Seqr
	NM_000137.2	Exon 2	CGCTCTGCATACAGGGCCAC	CCCAAGGCCACCTCCTACCA	Variant_Seqr
	NM_000137.2	Exon 3	TGGCCACCTTCAACTGTGGG	AAGGCTGCCAAGCCGTGACT	Variant_Seqr
	NM_000137.2	Exon 4	CCAGCCAGAAGGTGCCCACT	ATTTGCTGTGCAGGGAGCCA	Variant_Seqr
	NM_000137.2	Exon 5	TGCATTTGGTGCTGCCATTG	GGACTTGCCCTTCGATCCCA	Variant_Seqr
	NM_000137.2	Exon 6 and 7	TGAGCCATGTGTATGTGGCGA	GCTGCCGATGTGGCTGAAGA	Variant_Seqr
	NM_000137.2	Exon 8	AGTGGATTCATGCCAGGCCC	GTCAGCCGTGTCCCTAGCCA	Variant_Seqr
	NM_000137.2	Exon 9	GCACAGTGCCCTCAAAGGACC	GCCAGGGAAACCACAAGGGA	Variant_Seqr
	NM_000137.2	Exon 10	CCATCTCGGCCTGTCTGCTG	GCCACACACATCTCCAGGGC	Variant_Seqr
	NM_000137.2	Exon 11	GGTCACAAGTAGCTTTCAAATGGCA	CATGGAAGCTCCAGGCCCA	Variant_Seqr
	NM_000137.2	Exon 12	GGACTGGAGAGAGCTGGCTGG	CACGTGTCACTGCGCTGCTC	Variant_Seqr
	NM_000137.2	Exon 13	TCGGGACTCCCAGGTCTTGC	GTCTGCACAGGGCGTTTGCT	Variant_Seqr
	NM_000137.2	Exon 14	AAGGGCTCCTGCGTGCAACT	CCAGGAGGCCTGGGATGTCT	Variant_Seqr
VLCADD	NM_000018.3	Exon 1 and 2	GTGCACTGTGGACGATGAGTCAG	GAAGCGGTGACCTTTCCCCTAGT	Primer Designer
(ACADVL)	NM_000018.3	Exon 3, 4 and 5	GGGAAAGGTCACCGCTTCGC	CCAAGCCCCGAGTCATTCCTTA	Primer Designer
	NM_000018.3	Exon 5 and 6	CCGGTAAGGGAAGGGATAATCAGA	GGCAGGTGCTTAGAAGAGACCTT	Primer Designer
	NM_000018.3	Exon 7	TAGGTCAGGAACTGCCCTGT	TGGCCTAATCTGTGCCAAGC	Primer Designer
	NM_000018.3	Exon 8 and 9	GAACTGGATACTCCCAGGTGTT	TAAGCTCCCCCAACCCAAATTCA	Primer Designer
	NM_000018.3	Exon 9	CTGCCTCCCATTTCTCCCCTTCTC	CAGGGAGGAATGACGACCACTA	Primer Designer
	NM_000018.3	Exon 10	ATAAGGAGCGAAGGAGCAGTT	TTCTGCAGTCTCCCTAGGAGAGG	Primer Designer
	NM_000018.3	Exon 11	TTGTATGCAAAACCCATCCCTCT	GTTGTAGTCTGACCCGACTGG	Primer Designer
	NM_000018.3	Exon 12 and 13	CTTATCTTGGAGATCTGGGTGATGA	GAGCCACAAACAGCCGAAGAAT	Primer Designer
	NM_000018.3	Exon 14 and 15	TACAGGACGGTCTTCTGCAGAG	CTCAACTCCGGGTGGACAAGT	Primer Designer
	NM_000018.3	Exon 16 and 17	AACAGCTGAGGCGGTAGGCTTA	CTGCCTCCTCACCTCGAGAGAA	Primer Designer
	NM_000018.3	Exon 18 and 19	TCAGTAAGTGAGCTCTACACCATTC	AGGCCTTGGAGATGCTTTTGAAGTT	Primer Designer
	NM_000018.3	Exon 19 and 20	CAGGCAGGGAATGCCTGAGC	AGGCACAGTGCTGGGAACACTA	Primer Designer

Abbreviations: BKT: Beta-ketothiolase deficiency, ADA: Adenosine deaminase deficiency, BTD: Biotinidase deficiency, CACT: Carnitine-acylcarnitine translocase deficiency, CPT1A: Carnitine palmitoyl transferase 1 deficiency, CPT2: Carnitine palmitoyltransferase 2 deficiency, CTD: Carnitine transporter deficiency, GA1: Glutaric aciduria type 1, HCY: Cystathionine β-synthase deficiency, IVA: Isovaleric acidaemia, LCHADD: Long-chain acyl-CoA dehydrogenase deficiency, TFP:Trifunctional protein deficiency, MCADD: Medium-chain acyl-CoA dehydrogenase deficiency, PKU: Phenylketonuria, TYR: Tyrosinemia type, VLCADD: Very long-chain acyl-CoA dehydrogenase deficiency. AA: Amino Acid.

All primer sequences are designed to include the canonical splice sites. For most amplicons a minimum of +/- 5 base pairs from exon start/stop are covered. In some instances, primer design with these parameters could not be accomplish this due to difficult areas in the intronic areas 5' and/or 3' of the exons, which in the worst cases resulted in some amino acids being omitted (listed in the table as missing AA's). For ETFB: exon 2 +/- 4 bp included.[30] Wang, Y., Kelly M. A., Cowan T. M., and Longo N. A missense mutation in the OCTN2 gene associated with residual carnitine transport activity. *Human Mutation*, **2000**. 15, 238-245.

Table S2. Overview of the primer-pairs used for confirmation of diseases mainly sequenced using AmpliSeq panels.

Gene	Transcript	Covering	Forward primer	Reverse primer	Primer design
CACT	NM_000387.5	Exon 1	TGGCGCAGGGTCAGAAGAGA	GCTTTGCGCTCTGCCACG	Variant_Seqr
(SLC25A20)	NM_000387.5	Exon 2	GTGAAGGAGCGGCAGCTGTG	TCCAGCAGGTTAAGTGAATGCCA	Variant_Seqr
	NM_000387.5	Exon 3	TATTTTAACCCATGTCACGCTACCA	CCTTCAGTTTCTCCAGCATAATAGG	Primer designer
	NM_000387.5	Exon 4	GGGTGAAAGGACATAAACATGCACA	TGTGGCCAGACTCCCATTGC	Variant_Seqr
	NM_000387.5	Exon 5	CCCTGCAGACACCTGGGAAA	CCTGCTGGGTCTGTGACTCTGA	Variant_Seqr
	NM_000387.5	Exon 6	GTGCTGCTGGCACTCCTTGG	TTGAACCAAATCTGGTCACCCA	Variant_Seqr
	NM_000387.5	Exon 7	TGGGACAAATGCACAAATGCC	CTCCAAGGAGTGCCAGCAGC	Variant_Seqr
	NM_000387.5	Exon 8	TGCCCACTTCTAATGGCCAGG	GGCATTTGTGCATTTGTCCCA	Variant_Seqr
	NM_000387.5	Exon 9	TCAGCTCCTGGTGATCTGGTTGA	TTGATGTGGAGTGGGTGTGGG	Variant_Seqr
CPT2 (CPT2)	NM_000098.2	Exon 1	CAACTCCAGTTTCTGTCTTGCTCCA	GGGCGGAAACGGGTTCACTA	Variant_Seqr
	NM_000098.2	Exon 2	CCATTGATTCTGATTTGTCAGTCGC	GGTTCCTGGAGACCCAGCCA	Variant_Seqr
	NM_000098.2	Exon 3	TCTCCATGCCAAACTGCCTCC	GGCTGGCCATCATTCTTGAGC	Variant_Seqr
	NM_000098.2	Start exon 4 -> AA266	CAGGGAATTCTTCTCTCTGGAGGTTG	GGGCTCACAATGTTCCCATCTTG	Variant_Seqr
	NM_000098.2	Exon 4 (AA203 -> AA400)	GGGCCTACCTGGTCAATGCG	TCTGTGGAGTGACGGCAGGG	Variant_Seqr
	NM_000098.2	Exon 4 (AA410 -> AA526)	AAAGTGGACTCGGCAGTGTTCTG	AAAGGCCTCAGAGCACCTCTTTG	Variant_Seqr
	NM_000098.2	Exon 4 (AA410 -> end Exon 4)	CACGGTGCAGAAACTCAACTTCG	TGTGGCTGTTCAGACTGCTAGGG	Variant_Seqr
	NM_000098.2	Start Exon 5 -> AA627	TGCTCTGAAGGTTAGTCAGTTGGTGG	GGAAGAGACATTGCAGCCTATCCA	Variant_Seqr
	NM_000098.2	Exon 5 (AA557 -> end Exon 5)	GTTTGCTCTGCGGCATCTGG	TTCATGATGAGGAAGTGATGGTAGC	Variant_Seqr
GA2 (ETFA)	NM_000126.4	Exon 1	AGTGATCTTTGCAAGACCCCATAG	TTACCCCGAGGTCAGCTCAGT	Primer Designer
	NM_000126.4	Exon 2	AAGCATAATTCTCTGTTGCCATCT	GTGGTACAGTGATAGACACAAATAGC	Primer Designer
	NM_000126.4	Exon 3 and 4	AAATCCAGATTGGCTACATAAACAGT	TAATTCTTAACTGGCCCTTTTGCT	Primer Designer
	NM_000126.4	Exon 5	TGGTCCACTTAAAGCAATTGTATGG	TCCACAACTGTCACACTTGGTA	Primer Designer
	NM_000126.4	Exon 6	TGTAGGCAGAGGTCATCTCTTATT	TGGAGAAGGAAGCTTTGTTCATATTC	Primer Designer
	NM_000126.4	Exon 7	CCCAAACTTCCCAACTTCTTACAT	TGGAGGGTGTCACACTTTAACATAT	Primer Designer
	NM_000126.4	Exon 8	GAGGCCTTCTCCTCACTGAATGC	GGAGGTGACATGCTTAATATGGCAGT	Primer Designer
	NM_000126.4	Exon 9	GCTGGATTTAAGAACTGCTGGGC	GCATGCAGAAGAGTTAAGCCCAAA	Primer Designer
	NM_000126.4	Exon 10	CCCAGATCTACGTTAGTGTGAATGGC	CAGCATGTATGGGTTGTGCAGTG	Primer Designer

	NM_000126.4	Exon 11	CAAACACAGACACACAAACACACAATG	GCACTACAAAGGCTGTGGATTTGG	Primer Designer
	NM_000126.4	Exon 12	GGTTGATTAGGCTGGTGGAGACA	GCCCTCGTGCCTCAACAGTG	Primer Designer
GA2 (ETFB)	NM_001985.3	Exon 1	TTACGAGAAGACCCCCACCCA	GAAGTGTTTATAGGACCGGGAGGAT	Primer Designer
	NM_001985.3	Exon 2	CCCCCTCCCCAAGACCTTCA	ACCTCTCACTGTGGGTTACCATAAAT	Primer Designer
	NM_001985.3	Exon 3	GACTCTGTCCTCCCTGCTCAAAC	CCTGAGGATAGCAGCCAAGTCA	Primer Designer
	NM_001985.3	Exon 4	CCCAGCCTGCATTTCTATTGGA	CCCTGTTCTAGACAGAGGCCTGAA	Primer Designer
	NM_001985.3	Exon 5	GACCTCCTTTGGATCCTTCCCTCC	CTCACTGGCAGCATCAGGACATG	Primer Designer
	NM_001985.3	Exon 6	ACAGAGATAGATGTTGAGAGTCAGTTTTATTG	GTTCCACAGCCCTGTGAACA	Primer Designer
GA2 (ETFDH)	NM_004453.3	Exon 1	GCAGCAGAGTTCTTGCTTTCC	AGATAGCCTGAGAAAGCTGATGAGA	Primer Designer
	NM_004453.3	Exon 2	CCATAGTTAAGCCTGACATGAGCTA	ACTCTCCTCCACAAAATGAAAACAA	Primer Designer
	NM_004453.3	Exon 3	AGTATGTACTGGAACAGAACCTAAGA	AGATAGAGTTACATGAAAAGGGTTTCC	Primer Designer
	NM_004453.3	Exon 4 (AA160 -> 163 missing)	(AAGGAAATGCCAAAAGAAAATTAGGG	TGCATATTCACTATACCTTACCTGGAAGA)	Primer Designer
	NM_004453.3	Exon 5 (Start -> AA189)	GCCAATTCTTCCAGGTAAGGTATAG	TAACCAGGGTATACTTCAACACCAA	Primer Designer
	NM_004453.3	Exon 5 (AA171 -> End)	AGGGCTTCCAATGAATAATCATGGC	TCCAGAGTAAAGATAGGGAGGAAGA	Primer Designer
	NM_004453.3	Exon 6	AGAAGATGTTCACCTTCTAAGAAACC	ATGTACAGTTTGTAGCAAGACCTTT	Primer Designer
	NM_004453.3	Exon 7	TGCACTGTCTCTTCTACATCTGA	TCAAGATTCACTTCAAGCAACACT	Primer Designer
	NM_004453.3	Exon 8	GTGACATAAAAACATTTTTAAAATACTGCAGGAC	ACTGATGGTAATATAACTCTAGCAGCAGA	Primer Designer
	NM_004453.3	Exon 9 (Start -> 359)	TTGAGTTCCAGAGCACAAGGATTTC	TGAGAGCTCTGGCTCCGTATGC	Primer Designer
	NM_004453.3	Exon 9 (AA341 -> End)	ATCCATACCTGAGTCCATTTAGAGAGTT	GACACAGTTTTATAAACTCTACATACTTTTCCT	Primer Designer
	NM_004453.3	Exon 10	GCCTTTCCCTACAGCTCTAGAATAC	GCTACCACTGGTACTTTTCTACTTTT	Primer Designer
	NM_004453.3	Exon 11	AAACTTCACTCATCAGCTATCAAAGT	CTTCAACAATTTTGAGGGAAATGCT	Primer Designer
	NM_004453.3	Exon 12	GGCTAGTCATATTTCTTTGGTGTGA	CTGAACTAGGCTTAACAAAACCAGTA	Primer Designer
	NM_004453.3	Exon 13	CTCTATGGGTAAGCATTCAGAAAGCAA	CTTTGGCAAGTCAAATGTAAAGTCCAG	Primer Designer
HCS (HLCS)	NM_000411.6	Exon 4	CGAACTCCTGGGCTCCAAGC	TGTTGTCAGGTAGAGATGAGAATGCTG	Variant_Seqr
	NM_000411.6	Exon 5 (Start -> 157	GATGTTGGGTGCCTTTCCCG	TGCCAGAGGCTGTCCTGGAAT	Variant_Seqr
	NM_000411.6	Exon 5 (AA60 -> 243)	GGTGAAGGATGAAGACAGGCCC	GCCTGAGCAGGACGGTATGGA	Variant_Seqr
	NM_000411.6	Exon 5 (AA194 -> End)	AAACAGCCCGCAGCACACG	GGCCGACTGTGTGGACATTGA	Variant_Seqr
	NM_000411.6	Exon 6	TGAAGATGATTTCCAAACCCGAA	TGGGTGTGATGTGCTCACTTGG	Variant_Seqr

		Exon 6 (Start -> AA379)	GTGAGTGCCCTCAGTTTAC	GAGGAGTTAAGGCAGGAAC	Variant_Seqr
	NM_000411.6	Exon 7	GCACAACCACCTTCCATTCCC	TGCCAAATTACGACCATCTCATTTC	Variant_Seqr
	NM_000411.6	Exon 7 (Start -> AA475)	AGGAGACGCATCGTTGTGGG	TCTTGGATCCTGGTTTCTGAAGAGC	Variant_Seqr
	NM_000411.6	Exon 8	TCTTCCTCCATTCCAGGCGG	TCAGGCAGGTACACACGCCC	Variant_Seqr
	NM_000411.6	Exon 9	CCGAGAGCACTTTGCTGCCA	TCAAGGCACAAATGATATGCTCACA	Variant_Seqr
	NM_000411.6	Exon 10	TTTGGTGGTTCCCGTGGTCA	CCCAAGAGGCACTTCAATATCGG	Variant_Seqr
	NM_000411.6	Exon 11 (Stop codon missing)	TGAAGATGAGCCAGCACTGACC	CACGAAATCTGAAATGAGGACAAGGA	Variant_Seqr
	NM_000411.6	Exon 11 (AA620 -> End)	GACCAGTGCTCGGTCCCTCA	TGCATCAACGACCTCATCACAGA	Variant_Seqr
	NM_000411.6	Exon 12	CGTAAGTCCAAGCCACAGAGGG	TCCCTGCAGCGAGCCTCA	Variant_Seqr
HMG	NM_000191.2	Exon 1	ATGAAGAGTTGGAGAGGTCTGAAGT	GTGGAGCCAGCTTCGGAAGTC	Primer designer
(HMGCL)	NM_000191.2	Exon 2	CCAAACCTGTTGCACCACCATTC	TTCATATTGGTCTCCCTGGGAATTG	Primer designer
	NM_000191.2	Exon 3	TCACCTGAGACCTCTCAAAGCCA	TCTGCCCTTTCAGGCCATCA	Primer designer
	NM_000191.2	Exon 4	GTAAGAAAGTTGGCAGATGCCAGCAA	TCTCTGTCTGCTCTTGGTGATGAC	Primer designer
	NM_000191.2	Exon 5	GACCACTTGAGTCAGAGTCTAGC	AAAAGTGCTGAAAGGAGTTCACCT	Primer designer
	NM_000191.2	Exon 6	GGGAGAGGAACCTATGCGCTCA	CCAGCTCCTCTGGCACGTAGTT	Primer designer
	NM_000191.2	Exon 7	CACCATTGCCAGCTTGGAACC	CCCAGAAGTGTTCTGCCCAGG	Primer designer
	NM_000191.2	Exon 8	GCGTGTGGCCTCACTCAACC	TCAGGCAACAGACGATTGGGA	Primer designer
	NM_000191.2	Exon 8	CATCCACTTTTGTTCTCAGCTTCAG	GTTTGCAAGGCTCATCTCTGTG	Primer designer
	NM_000191.2	Exon 9	CACGTCCTCAGGCATTCAACTC	TGTCAAGGAAAAAGCCTGTTTATCT	Primer designer
MSUD	NM_000709.3	Exon 1	AGGCAGGCCTTGGCATTGAT	TGGCAGATCTTCCCTTCGGTG	Primer Designer
(BCKDHA)	NM_000709.3	Exon 2	CAGTTATTGACTTGCCAGCCACTTG	TGCGGGTGGAGGCACAGATA	Primer Designer
	NM_000709.3	Exon 3	CGGCGGAGTTTATAGATAAGTTGGA	TCAGGTCCATCCTTCTGCCCTAAG	Primer Designer
	NM_000709.3	Exon 4	CCTCTGGCAGTTCTAAGCAGTCT	ACAGACCCTGAGCCACTACACTTT	Primer Designer
	NM_000709.3	Exon 5	TCAGTCTGAACATCAGTCTTCCT	AACCCAGAATTCCACCCAGAAG	Primer Designer
	NM_000709.3	Exon 6	CCGTGGGTCATGTGAGTGTGAAT	ATAAGGAACATGCACGAGGCCAAG	Primer Designer
	NM_000709.3	Exon 7	CTCTCTGTCCTCTCCCTGCTCG	GTACGCTGAACTGTCGTCACT	Primer Designer
	NM_000709.3	Exon 8	CACTCCTCCTTCCCTAGTTCATC	CCAGTTTCTGAAATGGGCACCA	Primer Designer
	NM_000709.3	Exon 9	TCCATTGTCGAGGTGGGAACA	TGAATGTGCCCTCACCCAGC	Primer Designer
MSUD	NM_000056.4	Exon 1	CAGCTCGTCACGTCGCCTT	GCAAGGAGGTTCCAGAGAGTCAG	Primer Designer
(BCKDHB)	NM_000056.4	Exon 2	ATTAACAAGCTTCTCCAGGTCTGT	AATATCAGGACCACACACACTGTA	Primer Designer
	NM_000056.4	Exon 3 Start -> AA106)	ACAGACCCTCACAACAAAAGAATCA	TTTGTCTCGCAAGCCAACAG	Primer Designer

	NM_000056.4	Exon 3 (From AA104 -> End)	GAAGATGTTGCCTTTGGTGGAG	CATTTGAGAAAATATAGGCCAAAGGATTCT G	Primer Designer
	NM_000056.4	Exon 4	TCCTCTACCTGTTCTATACTTCTCCAT	CCCAATCCCAATCTATCTGCTTAAA	Primer Designer
	NM_000056.4	Exon 5	GAAAGACTTATTGTGCTATGGGAAAGA	AACTGCTAACCTCCCCTTCTTAAAT	Primer Designer
	NM_000056.4	Exon 6	AGTTAACAGAACATGGTAGACATCTGA	CCAAACATAAAATTTACACCTGAGGAC	Primer Designer
	NM_000056.4	Exon 7	GCAGTAATGTCATGGAGCCATAT	TAGCATCAGTAGCACCAGACTTAAT	Primer Designer
	NM_000056.4	Exon 8	ATTCTCCATGCAGATCAGTTCCT	CAATTCAACCCAAGGTGACTGCAT	Primer Designer
	NM_000056.4	Exon 9	TTTCTTTTCTACTGGGATTACAAACCA	TAACAAACAGAAAGTGTGGTCAGAA	Primer Designer
	NM_000056.4	Exon 10	ATCCCAGTTTGGCTTAGACAATAGA	AGCACATTATTTTCTTCTGAAAGTTGC	Primer Designer
MSUD (DBT)	NM_001918.3	Exon 1	AGAAAGTAAAACACCACTCCTGGAT	TCAGGTCCTACTGCAACATAACTTT	Primer designer
	NM_001918.3	Exon 2	CCGGCTAGAAATACAGAGTCAAC	AGTTTTAGTTCAGGAAGTCCAATGAC	Primer designer
	NM_001918.3	Exon 3	ACGTTTCTCAATTTAAATGGATTCCCA	AGCTGGGTCTCTCTTTGTATATCTT	Primer designer
	NM_001918.3	Exon 4	TCTATTTTGTGTTGAACTTGCTACCT	AACATTGCTTAGTACACCATCTGAAA	Primer designer
	NM_001918.3	Exon 5	ACAACTTCTCATTTCTCCCATGTATTT	GAGTGACATATCCACCAGGTACTA	Primer designer
	NM_001918.3	Exon 6	TGAACTTTCCCTTCAGTAAGACTTG	ACCTGATGGTTACCACATGCATAT	Primer designer
	NM_001918.3	Exon 7	CAAGCCTTGTTTGAAATGAATGAATCT	GATTAACTTCCAGAAGAGTGGCTTT	Primer designer
	NM_001918.3	Exon 7 (AA278-> End)	CATATTGCATGTTGGCAAAGCAA	CAGCCCTGAAGATACCTCATTTTGG	Primer designer
	NM_001918.3	Exon 8	GAGGGTAAGTAGAGTGGGTAACAT	GCCTGAAGGTAACATTGGCAGTAA	Primer designer
	NM_001918.3	Exon 9 and 10	TTCATTGTGTTTAGTCCCTGAATTTGC	TGGCAGTGAAGGTTGATCCTTTCTT	Primer designer
	NM_001918.3	Exon 11	TCTTGCCAGTTTCAAGCCATTGAC	GGGTTTGCCTGATCTTACACCATTG	Primer designer
	NM_001918.3	Exon 11 (UTR)	ACTGTCTGTATATGGCTACCTGTC	CGTATGATTAAGGATCTAAGGCACAA	Primer designer
MMA (MMUT)	NM_000255.4	Exon 2 (Start -> AA41)	CTTTTTAGCCAGGGCAGCCCATTC	CCATGAGGAGTGTGACATAGTCA	Primer designer
	NM_000255.4	Exon 2 (AA25 -> End)	CACTTATCTTTTTGACCACAGAAGTT	TGAGGCAGGTAAAAGAATCATCAG	Primer designer
	NM_000255.4	Exon 3 (Start -> AA181)	CCATTCATAGTCATGGAAACTGACA	TTGCCATATATGAACAGCCAGATC	Primer designer
	NM_000255.4	Exon 3 (AA155 -> End)	TCTGTTGCCCAGATTCCTGCAAGTA	GATTCAGACAACCCTCGAGTTCGT	Primer designer
	NM_000255.4	Exon 4	TTGGCTTTTTCTCTCATTATCACTCA	AAGTAGGTACAGTCCTGATGATGGT	Primer designer
	NM_000255.4	Exon 5	GCTTCTTAATTCTATTTCCTGCTTGTG	AGGGCATAGGAAGAGATTCTTTCAT	Primer designer
	NM_000255.4	Exon 6 (Start -> AA389)	TGGCAAACCCAAAGCTTCATCA	CCAATGACTTGCCCAAATCACAA	Primer designer
	NM_000255.4	Exon 6 (AA393 -> End)	TGTAAATTCTGAAAACAAAGTTGCAAAGTG	AGTCTTTGCACACAAATTCTTTTGATGAA	Primer designer
	NM_000255.4	Exon 7	TGTGTGCATCCATGTATGTGAAAAT	ATTTTCTCCCAAGACTTAAGAGGTTT	Primer designer

	NM_000255.4	Exon 8	AGTTTCACAGAGGTAGATATCATGGT	CCCTTCTCAGATTGGGATTTGC	Primer designer
	NM_000255.4	Exon 9	GCCATTATTTTCTTTTGGGCTCACA	GGTCAGCACTATTATTGCCCTTCAT	Primer designer
	NM_000255.4	Exon 10	GTTGTATGTAAGGAAATTAAGCTCCCA	AGCAACCTTCAGGTACTTGTCTA	Primer designer
	NM_000255.4	Exon 11	AGTAAATACTTTTGAAATTCCCCCCAA	ATGGCTTTAATGGAGCTCTCAATG	Primer designer
	NM_000255.4	Exon 12	GATTCCCATCACAGTACTAGAAAAATAGAGATAAA	CCAGGGTTTTTATAGTCATTATCTAGTAATA AACA	Primer designer
	NM_000255.4	Exon 13	GCTTTCAAGGAAAGTACAAATCAGGTA	ATGGCTTAAGGCTCAGAAGTTCTC	Primer designer
MMAB	NM_052845.3	Exon 4	TGAAGAAATGGAGCTCACAGAGGG	TGCGGGTCAGAGCTCAGGAT	Primer designer
(MMAB)	NM_052845.3	Exon 7	TCAGAGATGGCCCTGCTGTACC	GCAGGTTCAGTGACAGACACACAT	Primer designer
PA (PCCB)	NM_00117801 4.1	Exon 1	CTTACTCCCAAGCAGGTTCTGTAG	ACAGGTGCTTCTGCTGGTTATAC	Primer designer
	NM_00117801 4.1	Exon 2	CCTTCAAGCCCTCCCATGAACA	TGCTTACATGAAGGAGGACGTATAA	Primer designer
	NM_00117801 4.1	Exon 3	TCATTGAGGCATAGTGGCCAAACTC	CACCCACTGCACTCTCTCTAGT	Primer designer
	NM_00117801 4.1	Exon 4	AGCCCTTTACATCTGAACATAATGAAG	TTGCAGTTCTGAAGGAAAACCTATC	Primer designer
	NM_00117801 4.1	Exon 5	TCTATTTCTTCCTCCCACTGGGTTT	GGAGGGTTCAGGCTTTGCCA	Primer designer
	NM_00117801 4.1	Exon 6	AACACTGTGGTATTTTGTGAATGTCG	TTCATATTTCCTCTCCCTTGCTTTC	Primer designer
	NM_00117801 4.1	Exon 7	TTAGGTCTGTTGCCTCCTGTATAGA	ACAGTCATCTATCTGGTCAAAGGA	Primer designer
	NM_00117801 4.1	Exon 8	TCCTCATTCAGCCACGTCACTA	ACCATTCCAGCAACTAGCTCTAAA	Primer designer
	NM_00117801 4.1	Exon 9	GTCTGCTACTGACATGCCCTAGA	AGGCCAGACCAAAAAAAACTTGAAT	Primer designer
	NM_00117801 4.1	Exon 10	ACGTGTCACCCCATTTCCTTTCC	TCTGGCCTCACTCTAGACTCTGT	Primer designer
	NM_00117801 4.1	Exon 11	AACAAACTTCATTGGTGTGTCCAT	ACAACCATTTAGATTTTTTCCTCTGGT	Primer designer
	NM_00117801 4.1	Exon 12 (Missing AA434 -> AA453)	CTTTGGATGGCTGCTGAGGACAA	TGCCTCAGCAAATGCGTAGAGAA	Primer designer
	NM_00117801 4.1	Exon 13	GGAAAGACCTCACAGCTGAGAGT	TGAAAGACCCCAGGAAACCAGGTAA	Primer designer
	NM_00117801 4.1	Exon 14	TTGTCCCAATTTTACCTGGTTTCCT	CAACCCTTTGCCCCCTTTACTT	Primer designer

NM_001178014.1	Exon 15	TATGGCCTTTAGAGATGGCACTT	TGGCAATGTTGTTTTAATTGTGGTT	Primer designer
NM_001178014.1	Exon 16	ATCAGGTTGGGCACTGCTTATACT	AGCTCTAGATGATAACACTGTGTACT	Primer designer

Abbreviations: GA2: Glutaric aciduria type 2, HCS: Holocarboxylase synthetase deficiency, HMG: 3-hydroxy-3-methyl-glutaryl-CoA-lyase deficiency, MSUD: Maple syrup urine disease, MMA: Methylmalonic academia, PA: Propionic acidaemia. AA: amino acid

All primer sequences are designed to include the canonical splice sites. For most amplicons a minimum of +/- 5 base pairs from exon start/stop are covered. In some instances, primer design with these parameters could not be accomplish this due to difficult areas in the intronic areas 5’ and/or 3’ of the exons, which in the worst cases resulted in some amino acids being omitted (listed in the table as missing AA’s).
